# Deterioration of Portland Cement Pervious Concrete in Sponge Cities Subjected to Acid Rain

**DOI:** 10.3390/ma14102670

**Published:** 2021-05-20

**Authors:** Longxin Gao, Yong Lai, Mohammad Rashadul Islam Pramanic, Wuman Zhang

**Affiliations:** 1 School of Transportation Science and Engineering, Beihang Univerisity, Beijing 100191, China; SY1813202@buaa.edu.cn (L.G.); pramanic@buaa.edu.cn (M.R.I.P.); 2 China Airport Construction Group Co., Ltd., Beijing 100101, China; laiyong_@126.com

**Keywords:** Portland cement pervious concrete (PCPC), acid rain, wet-dry cycles, deterioration

## Abstract

The deterioration of Portland cement pervious concrete (PCPC) subjected to wet-dry cycles in the simulated acid rain solution was investigated; 4% silica fume (SF) and 8% fine aggregate (FAG) were used to replace part of cement and the coarse aggregates (weight by weight), respectively. The wear resistance, the compressive, and flexural strength of PCPC were measured. The results show that after 12 wet-dry cycles in acid rain solution the compressive strength and the flexural strength of control PCPC are decreased by 30.7% and 40.8%. The final compressive strength of PCPC with 4% SF and PCPC with 8% FAG is increased by 6.9% and 30.3%, and the final flexural strength is increased by 25.4% and 72.3%, respectively. The wear loss of PCPC is decreased by 58.8% and 81.9% when 4% SF and 8% FAG is added to PCPC, respectively. The microstructures of PCPC with wet-dry cycles are also discussed.

## 1. Introduction

Portland cement pervious concrete (PCPC) can be used to reduce the urban waterlogging problems [1], the heat island effect [2,3], and the traffic noise [4]. The construction of the sponge cities in China has promoted the application of PCPC. Usually, PCPC has a lower strength, a higher voids ratio, and a higher water permeability coefficient [5,6]. The higher permeability is typically achieved by using little to no fine aggregate [7,8,9].

However, industrialization and urbanization bring serious acid rain problems to the world. More than one-third of China’s territory is covered by the acid rain [10]. Once the cementitious materials contact the acid substance, hydrogen ions (H^+^) in the acid solution react with Ca(OH)_2_ and the external calcium carbonate in the hydrated cement paste, which will cause the dissolution of cement hydration products. In China, the excessive emission of SO_2_ is the main reason for the acid rain, and the anions in the rain are mainly sulfate ions (SO_4_^2−^) [11]. SO_4_^2−^ in acid rain can also cause a corrosion damage to concrete. Usually, sulfate will react with 3CaO∙Al_2_O_3_ to form ettringite and gypsum, and the volume expansion of ettringite will damage the internal structure of concrete [12]. Zhou et al. [13] reported that the H^+^ dissolved corrosion occurred in the corroded area of concrete, the SO_4_^2−^ swelling corrosion mainly occurred in the severely corroded area.

Some papers have studied the degradation mechanism and the failure products of cementitious materials subjected to an acid rain. Chen et al. [10], Xie et al. [14], Su et al. [15], and Zhou et al. [16] found that the coupling effects of hydrogen ions and sulfate ions led to the corrosion of concrete in acid rain region, and the deterioration began from the outside to the inside until it was completely degraded. Fan and Luan [17] concluded that the porosity of concrete exposed to an acidic environment increased slightly during the initial exposure period and gradually decreased with the prolongation of the exposure period. The lower the pH value of the acid rain, the more significant changes of concrete porosity occurred. Wang et al. [18] found that the freeze–thaw damage in North China aggravated the deterioration caused by acid rain, and the deterioration increased with increasing acid rain acidity and the external loading. Kanazu et al. [19] reported that there was an approximately linear relationship between the erosion depth of the simulated acid rain and the total rainfall. Dias et al. [20] observed that the continuous acid rain exposure led to the deterioration of asbestos cement, accompanied by carbonization and leaching, which increased the porosity and reduced the strength of the thin fiber–cement sheets. Derry et al. [21] found that the properties of concrete exposed to the atmosphere were related to the continuous hydration of cement and the reaction products of atmospheric acid with cement components. Gay et al. [22] found that the surface solution of the material was always alkaline, even when the pH of the corrosion solution was as low as 1. Guo et al. [23] found that pervious concrete had a similar corrosion rate with those of ordinary concrete and mortar. The weight of specimen was an unsuitable index to evaluate the acid rain corrosion. Fan et al. [24] observed the damage index corresponding to the change of relative compressive strength was more suitable to describe the deterioration of concrete eroded by acid rain.

Several researchers have offered solutions to eliminate or control the acid rain’s effects. Zivica and Krizma [25] observed that the acid resistance of slag cement was significantly improved when SF activator was used. Concrete containing SF had a lower calcium and silicon ion leaching performance in a hydrochloric acid at pH 5.06 and a rainwater at pH 6.65 [26,27]. Bakharev et al. [28] found that alkali activated slag concrete had higher acid resistance than cement concrete with a similar grade. Lu et al. [29] also reported that the proper use of fly ash and SF in recycled aggregate concrete would significantly enhance the mechanical properties and the acid rain resistance. Preetham et al. [30] reported the coat of colloidal vehicular soot dispersed in ethanol medium improved the acid resistance of mortar.

## 2. Research Significance

Compared with the conventional concrete, the coarse aggregates in PCPC are only wrapped by a very thin hardened cement paste, which causes a larger porosity and a lower strength. Therefore, PCPC is currently used in the sidewalks, parks, parking lots, and other places. PCPC has hardly been used in the actual automobile roads. In addition, more than one-third of China’s territory is covered by acid rain. The erosion effect of acid rain on PCPC may be more serious. In order to realize the application of PCPC on the pavements with the vehicle loading, both the strength and the acid rain resistance of PCPC need to be improved.

In the study, the wet-dry cycles were used to simulate the action of acid rain. The addition of SF and FAG was tried to enhance the mechanical properties and the acid rain resistance of PCPC. The weight change, compressive strength, flexural strength, and abrasion resistance of PCPC subjected to acid rain cycles were investigated. The microstructures were also discussed to reveal the deterioration. The results are expected to promote the application of PCPC in the construction of the sponge cities in the acid rain region.

## 3. Experiment Detail

### 3.1. Materials

The chemical composition of Portland cement of 42.5 grade is shown in Table 1. SF with a specific surface area of 19,500 m^2^/kg is used. The SiO_2_ content in SF is 95%. The particle size range of coarse aggregates with an apparent density of 2631 kg/m^3^ is 4.75–9.5 mm. The fineness modulus of river sands with an apparent density of 2650 kg/m^3^ is 2.54. The fineness modulus is an index indicating the gradation and the coarseness of sands. The larger the fineness modulus is, the coarser the sand is. A superplasticizer is used to improve the workability.

The long-term exposure experiment can better simulate the corrosion of concrete by acid rain. However, it will take a long time to reveal the deterioration [19]. Therefore, the accelerated experiments are often used. The acid rain usually contains H^+^, SO_4_^2−^, and NO_3_. Wang et al. [11] found that acid rain in China should concern the mainly impact of sulfuric acid because the contribution of SO_4_^2−^ to the acidity of acid rain was much higher than that of NO_3_^−^. The acid rain with a pH value between 3.0 and 5.0 will seriously affect the mechanical properties and the durability of concrete [31]. In this study, the sulfuric acid and the nitric acid are used to simulate the acid rain with a pH value of 4.0, the molar ratio is 9:1. The water with a pH value of 7.0 is also used as another comparable medium in wet-dry cycles.

### 3.2. Specimens Preparation

The mixing proportions of PCPC are listed in Table 2. The water:cement ratio was 0.28 and the target void ratio was 20%. SF and FAG were used to replace part of cement and coarse aggregates (weight by weight), respectively. PCPC specimens were cast according to CJJ/T135-2009 [32].

The compressive strength was determined by three cubes with a size of 100 mm. Cube with a size of 150 mm was used to measure the surface wear resistance. The flexural strength was measured by three prisms. The size of the prism was 100 mm × 100 mm × 400 mm. The specimens were demolded and cured for 28 days under the standard condition.

### 3.3. Test Methods

#### 3.3.1. Strength

The compressive strength and the flexural strength of PCPC were determined according to GB/T 50081-2019 [33]. The compressive strength was obtained by three 100 mm cubes, and the flexural strength was obtained by three prisms.

#### 3.3.2. Abrasion Resistance

The abrasion resistance was measured according to a JTG E30-2020 [34]. The load of the pressure head was 200 N. The rotation speed of horizontal pallet was 17.5 ± 0.5 r/min and the transmission ratio between the spindle and the horizontal pallet was 35:1. The wear loss was used to evaluate the abrasion resistance.

#### 3.3.3. Water Permeability Coefficient

**Figure 1:**
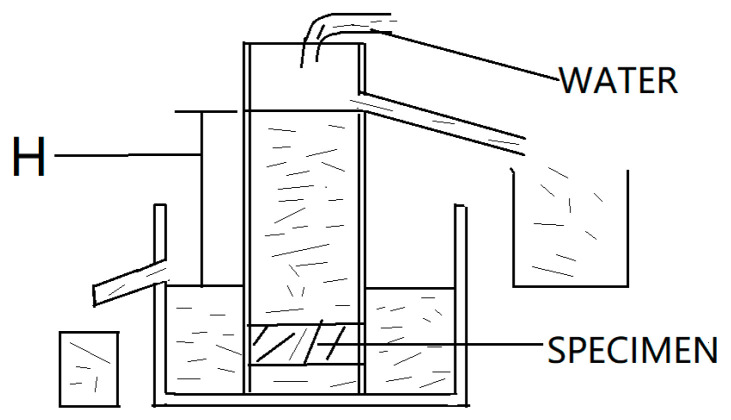
Schematic diagram of permeability measurement setup.

The water permeability coefficient was measured according to CJJ/T135-2009 [32]. Figure 1 shows the schematic diagram of the permeability measurement setup.

The water permeability coefficient is calculated by Equation (1).

(1) K=Qd/AtH

where *K* is the water permeability coefficient (mm/s), *A* is cross-sectional area of the specimen (mm^2^), *t* is the experiment time (s), *H* is the difference of water head (mm), *Q* is the seepage quantity at the time *t* (mm), and *d* is the height of the specimen (mm).

#### 3.3.4. Wet-Dry Cycles

In each wet-dry cycle, the specimens were completely immersed in acid rain solution for 3 days. Then, they were dried at 80 °C for 1 day. The pH value of the solution was measured and adjusted every day to ensure its stability; the solution was refreshed after each cycle. The weight change of the prism specimens was measured every cycle. The total number of the specimens in wet-dry cycles is shown in Table 3.

## 4. Results and Discussion

### 4.1. Weight Change

**Figure 2:**
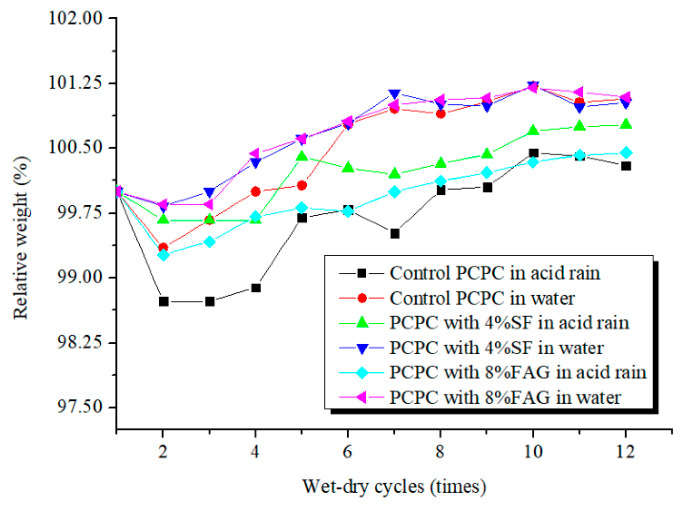
The relative weight of PCPC with wet-dry cycles.

Figure 2 shows the relative weight of the specimens. It can be seen that the relative weight first decreases and then increases with the number of wet-dry cycles increase. For control PCPC with two wet-dry cycles in acid rain and the water, the relative weight is decreased by 1.3% and 0.6%, respectively. The surface particles of sand (quartz) peel off from the specimens soaked in acid rain or in water, which causes the initial decrease of the relative weight of PCPC. The relative weight of the specimens with 12 wet-dry cycles in water is increased by 1.1%, and the weight of the specimens with 12 wet-dry cycles in acid rain is similar to that before the test. The increase of the relative weight is mainly attribute to the continue hydration and also the carbonatation of cement after the specimens absorb water [35]. In addition, the reaction products of sulfuric acid, nitric acid and calcium hydroxide have an early filling effect on the pores of concrete [36,37], which also slightly increases the weight of the specimens. However, if PCPC is subjected to more wet-dry cycles in acid rain solution, it is foreseeable that the acid rain will damage the hydrated cement paste, which will cause the spalling of PCPC and the reduction of the specimen’s weight. It also can be found that the relative weight of PCPC in acid rain solution is always lower than that of PCPC in water, which indicates that PCPC is corroded by the acid rain solution.

The relative weight of 4% SF PCPC or 8% FAG PCPC has a similar trend to that of the control PCPC. However, the change magnitude of the relative weight is smaller than that of the control PCPC, which indicates that the addition of 4% SF or 8% FAG tends to improve the acid resistance of PCPC. Usually, the hydration products of cement can remain stable in an alkaline pore solution [10]. However, calcium hydroxide will react with the acid solution followed by the acid dissolution of C–S–H when hardened cement paste is subjected to the acid solution, as shown in Equations (2)–(6) [38].

H^+^ + Ca(OH)_2_ → Ca^2+^ + 2H_2_O (2)

H^+^ + C–S–H → Ca^2+^ + SiO_2_∙nH_2_O (3)

H^+^ + C–A–H → Ca^2+^ + Al_2_O_3_∙nH_2_O (4)

2H^+^ + CaCO_3_ → Ca^2+^ + H_2_CO_3_ (5)

CO_2_ + Ca(OH)_2_ → CaCO_3_ + H_2_O (6)

The addition of SF will increase the Si/Ca and refine the pore structures of the hydrated cement paste, which finally increases the content of C–S–H and decreases the content of the Ca(OH)_2_ [39,40]. FAG can also improve the bonding interface between the hydrated cement paste and the coarse aggregates, so that the hardened cement paste will have a higher density and a better bonding strength, which finally increases the acid rain resistance. Similar results have been reported in [25,26,27,30].

### 4.2. Mechanical Properties of PCPC

#### 4.2.1. Strength of Control PCPC

**Figure 3:**
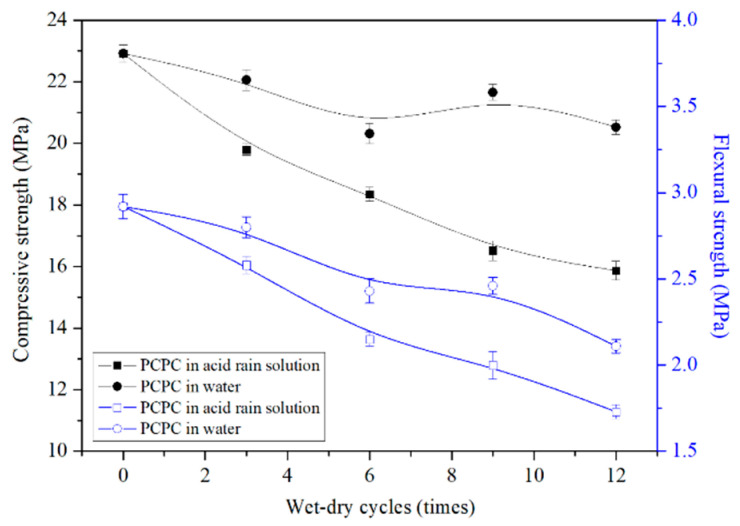
Strength of control PCPC.

Figure 3 shows the compressive and flexural strength of PCPC. The compressive strength and the flexural strength have a decreasing trend with the increase of the wet-dry cycles in the acid rain solution or in water. The strength reduction of the control PCPC in the acid rain solution is more significant than that of the control PCPC in water. The compressive and flexural strength of the control PCPC with 12 wet-dry cycles in the acid rain solution are decreased by 30.7% and 40.8%, respectively. Many researchers have found the similar results [14,20,24,30].

The strength reduction of the control PCPC subjected to wet-dry cycles in acid rain solution can be clearly explained by the corrosion of acid solution to the hardened cement paste. The neutralization reaction between the calcium hydroxide and the acid rain solution will reduce the alkalinity of the pore solution when PCPC is located in the acid rain environment. The low alkalinity of pore solution may lead to a decrease in the stability of C–S–H or even the degradation of C–S–H [10]. In addition, the dissolution of Ca(OH)_2_ and the decalcification of C–S–H decrease the Ca/Si ratio [37,41] and increase the porosity of the hardened cement paste, which causes concrete to be more easily damaged [38]. This process is a more serious damage to PCPC with a low bonding strength between the coarse aggregates and the hardened cement paste. Davis et al. [42] and Mori et al. [43] found that the SO_4_^2−^ could also accelerate the penetration of the H^+^ into concrete. The calcium hydroxide and C–S–H lose Ca^2+^ to form gypsum, and the later reaction between gypsum and the hydration products may also generate ettringite with a significant expansion in volume, which will decrease the strength of concrete.

Usually, PCPC subjected to the wet-dry cycles in water will show a periodic shrinkage and expansion deformation [44], which causes cracks in the hydrated cement paste and finally decreases the strength, especially for PCPC with the coarse aggregates only wrapped by a very thin hardened cement paste.

#### 4.2.2. Strength of 4% SF PCPC

**Figure 4:**
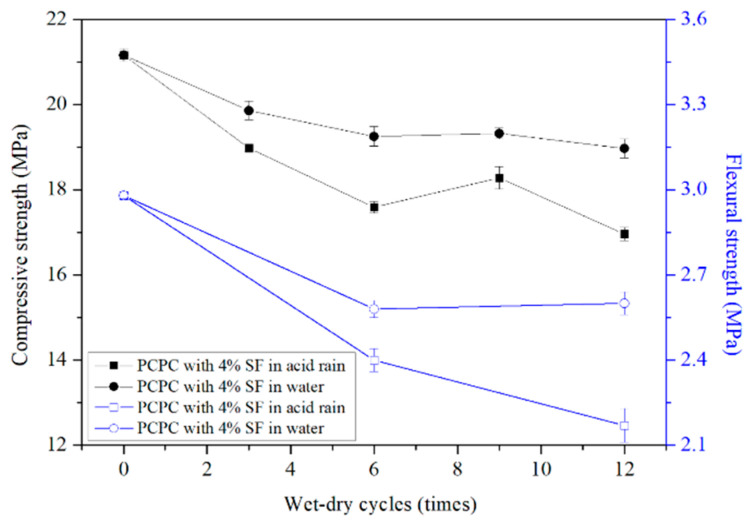
Strength of 4% SF PCPC.

Figure 4 shows the strength of 4% SF PCPC, and it is clear that the compressive and flexural strength of 4% SF PCPC under 12 wet-dry cycles in acid rain solution were decreased by 20.3% and 27.2%, respectively. However, the percentage of the strength reduction of 4% SF PCPC is lower than that of the control PCPC (30.7% and 40.8%). Moreover, the final compressive strength and the flexural strength are higher 6.9% and 25.4% than that of the control PCPC. The results indicate that the addition of 4% SF can improve the residual strength of PCPC subjected to acid rain attack [30].

Among the cement hydration products, the calcium hydroxide is the most easily corroded by the acid solution. The reaction between SF and Ca(OH)_2_ increases the content of C–S–H and decreases the content of the Ca(OH)_2_ [39,40]. In addition, the hydration of SF (as shown in Equation (7)) could refine the pores of the hardened cement paste, which is more difficult for hydrogen ions to penetrate into the hardened cement paste. These two actions of SF ultimately improve the acid resistance of PCPC [45,46].

SiO_2_ + 1.7Ca(OH)_2_ + 2.3H_2_O → CaO∙1.7SiO_2_∙4H_2_O (7)

**Figure 5:**
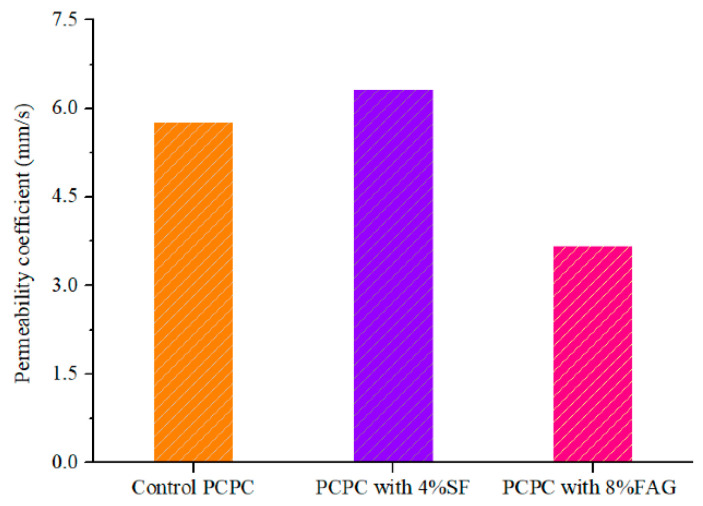
Water permeability of PCPC without wet-dry cycles.

For PCPC without silica fume, cement paste is easy to segregate, bleed, and accumulate on the bottom of PCPC during the casting process. The hardened cement paste will block some voids. For PCPC with SF, SF improves the cohesion of the cement paste [47] which can fully cover the coarse aggregates. SF reduces the segregation and causes the formation of more connected macroscopic voids in PCPC [48]. Water quickly passes through the macroscopic voids in PCPC with silica fume, which increases the water permeability of PCPC (as shown in Figure 5). Compared with the control PCPC, the permeability coefficient of 4% SF PCPC is increased by 9.5%.

#### 4.2.3. Strength of PCPC with 8% FAG

**Figure 6:**
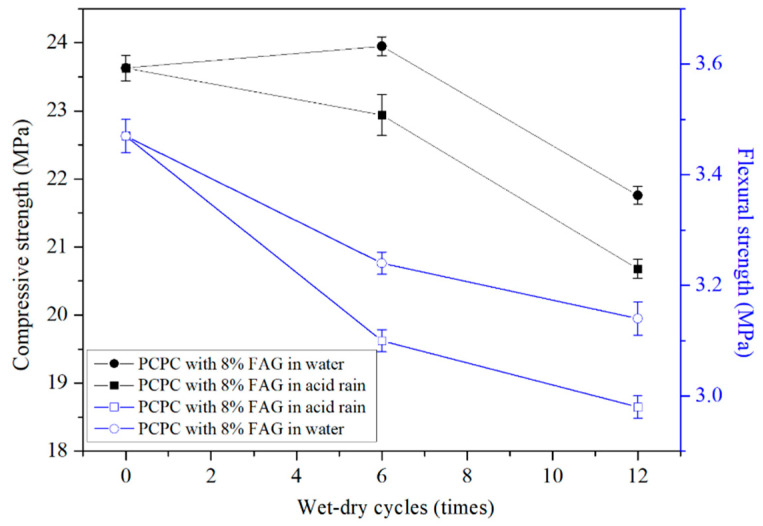
Strength of PCPC with 8% FAG.

The compressive strength and the flexural strength of 8% FAG PCPC are shown in Figure 6. It is clear that the addition of FAG improves the strength of PCPC. The similar result was observed by Alessandra et al. [49]. The compressive and flexural strength of the control PCPC increase by 3.0% and 18.8%, respectively.

However, the strength of 8% FAG PCPC also decreases with the increase of the wet-dry cycles. The compressive and flexural strength of 8% FAG PCPC under 12 wet-dry cycles in acid rain solution are decreased by 12.5% and 14.1%, respectively. The percentage of the strength reduction of 8% FAG PCPC is lower than that of the control PCPC (30.7% and 40.8%). The final compressive strength and the final flexural strength are higher 30.3% and 72.3% than that of control PCPC with 12 wet-dry cycles in the acid rain solution. The results show that 8% FAG can significantly improve the residual strength of PCPC subjected to the acid rain attack. FAG could reduce the void ratio of the hydrated cement paste and increase the apparent density of PCPC [50,51]. The apparent density of PCPC without wet-dry cycles is raised from 1840 kg/m^3^ to 2020 kg/m^3^ when 8% FAG is used to replace the coarse aggregates (weight by weight). The increase of the apparent density improves the acid rain resistance, which leads to a certain loss of the water permeability, as shown in Figure 5. Compared with the control PCPC, the permeability coefficient of 8% FAG PCPC is decreased by 36.5%. However, the permeability coefficient still meets the requirement of PCPC in the specification.

### 4.3. Abrasion Resistance

**Figure 7:**
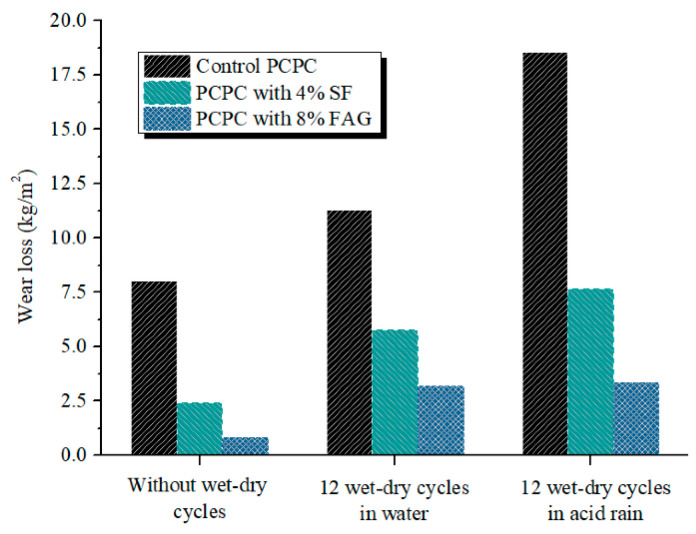
Wear loss of PCPC after the abrasion test.

As a road material, PCPC will be subjected to a long-term abrasion [52]. However, the low binding strength between the coarse aggregates and the hardened cement paste easily causes the loosening and the peeling of PCPC surface. In this part, PCPC is subjected to 12 wet-dry cycles followed by the abrasion test. Figure 7 shows the wear loss of PCPC during the abrasion test.

It can be seen that the wear loss of PCPC with wet-dry cycles in the acid rain solution or in water is significantly higher than that of PCPC without the wet-dry cycles probably due to carbonatation of portlandite. For example, the wear loss of the control PCPC with 12 wet-dry cycles in acid rain solution is ~2.3 times that of the control PCPC without wet-dry cycles. Xie et al. [14] also reported that the coupling actions of H^+^ and SO_4_^2−^ could corrode the microstructure of concrete and decrease the bonding strength between the matrix and the aggregates. Chen et al. [10] found the surface layer of cementitious materials subjected to continuous acid rain attack became softened, followed by a great deal of damage. Therefore, the wet-dry cycles in acid rain decrease the abrasion resistance of PCPC.

However, 4% SF and 8% FAG decrease the wear loss of all PCPC with and without wet-dry cycles in the acid rain or in water. The wear loss of PCPC subjected to 12 wet-dry cycles in acid rain solution is decreased by 58.8% and 81.9% when 4% SF and 8% FAG are used to replace part of cement and the coarse aggregates, respectively. On the other hand, the addition of 4% SF and 8% FAG improve the abrasion resistance of PCPC subjected to wet-dry cycles in acid rain or in water. The results are consistent with the effect of 4% SF and 8% FAG on the strength of PCPC.

**Figure 8:**
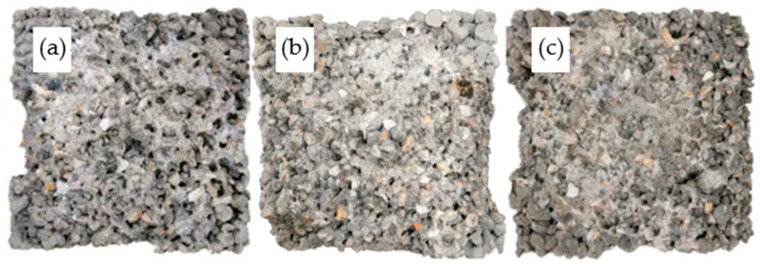
Surface changes of control PCPC after abrasion test. (**a**) Without wet-dry cycles, (**b**) 12 wet-dry cycles in water, and (**c**) 12 wet-dry cycles in acid rain.

**Figure 9:**
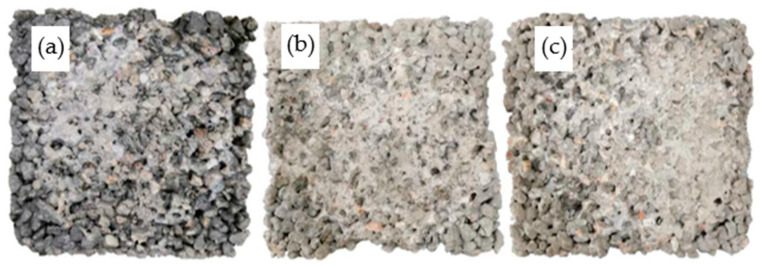
Surface changes of 4% SF PCPC after abrasion test. (**a**) Without wet-dry cycles, (**b**) 12 wet-dry cycles in water, and (**c**) 12 wet-dry cycles in acid rain.

**Figure 10:**
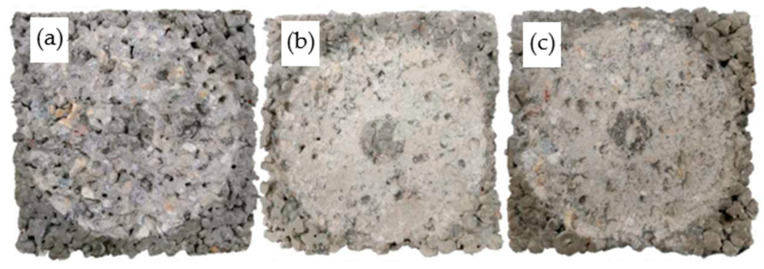
Surface changes of 8% FAG PCPC after abrasion test. (**a**) Without wet-dry cycles, (**b**) 12 wet-dry cycles in water, and (**c**) 12 wet-dry cycles in acid rain.

The surface changes of PCPC subjected to 12 dry–wet cycles followed by abrasion test are given in Figure 8, Figure 9 and Figure 10. The surface changes of PCPC are consistent with the wear loss. Especially for the control PCPC with wet-dry cycles in the acid rain solution, the coarse aggregates at the edge of PCPC also spall off during the abrasion experiment except the wear loss of the surface hardened cement paste. The specimen’s contours have become incomplete, as shown in Figure 8c.

For PCPC mixed with 4% SF or 8% FAG, only the surface-hardened cement paste is abraded and the coarse aggregates at the edge do not spall off. The specimen’s contours also remain relatively complete, as shown in Figure 9 and Figure 10. These results can be explained by the improvement of the microstructure of the hardened cement paste mixed with SF [53], probably due to calcium silicates formed, much less soluble than portlandite or other compounds, the increase in the binding points between the coarse aggregate particles and the decrease in the porosity of the hardened cement paste mixed with FAG [54,55].

### 4.4. Microstructures

**Figure 11:**
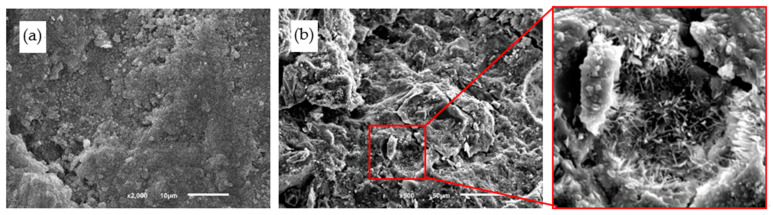
Microstructures of control PCPC. (**a**) Without wet-dry cycle and (**b**) with 12 wet-dry cycles in acid rain solution.

The microstructures of PCPC are observed to reveal the corrosion mechanism of acid rain. Compared with control PCPC without wet-dry cycle (as shown in Figure 11a), it is clear that the microstructures of control PCPC with 12 wet-dry cycles in acid rain solution are corroded by the acid rain solution. In Figure 11b, more crystals are observed in the pore of control PCPC with 12 wet-dry cycles in acid rain solution. H^+^ will cause a series of chemical reactions, as shown in Equations (2)–(6).

Equations (2)–(6) cause the continuous changes of cement hydration products, which causes a serious damage to the microstructures of the hydrated cement paste [10]. In addition, the reactions (as shown in Equations (8) and (9)) between SO_4_^2−^ in the acid rain and cement will produce gypsum and ettringite with a significant expansion in volume, which leads to an expansive damage to the matrix. The combing actions of the chemical reaction and the physical expansion will obviously deteriorate the properties of PCPC [56,57].

SO_4_^2−^ + Ca^2+^ + 2H_2_O → CaSO_4_∙2H_2_O (8)

4CaO∙Al_2_O_3_∙12H_2_O + 3CaSO_4_ + 20H_2_O → 3CaO∙Al_2_O_3_∙3CaSO_4_∙31H_2_O + Ca(OH)_2_ (9)

**Figure 12:**
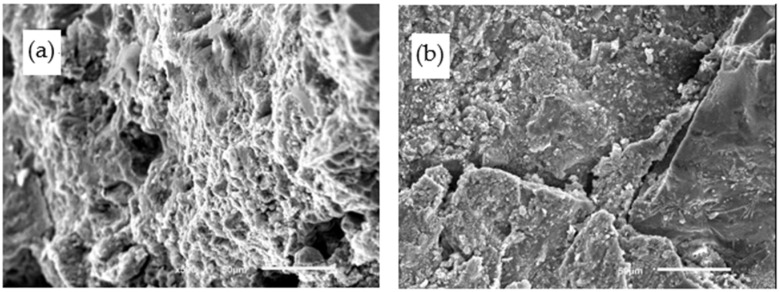
Microstructures of 4% SF PCPC. (**a**) Without wet-dry cycle and (**b**) with 12 wet-dry cycles in acid rain solution.

The microstructures of 4% SF PCPC are given in Figure 12. The addition of SF can enhance the interfacial transition zone by reducing its thickness and increasing its strength [58]. In addition, SF increases Si/Ca ratio in the matrix, which refines the pore structures and improves the microstructures and the durability of hydrated cement paste [10,40]. Adil et al. [48] reported that PCPC with 5% SF had a good strength and durability. However, microcracks are also observed in 4% SF PCPC (as shown in Figure 12b), which indicates that PCPC with 4% SF is still corroded by the acid rain solution, although the addition of 4%SF improves the final strength of PCPC with 12 wet-dry cycles in acid rain solution.

**Figure 13:**
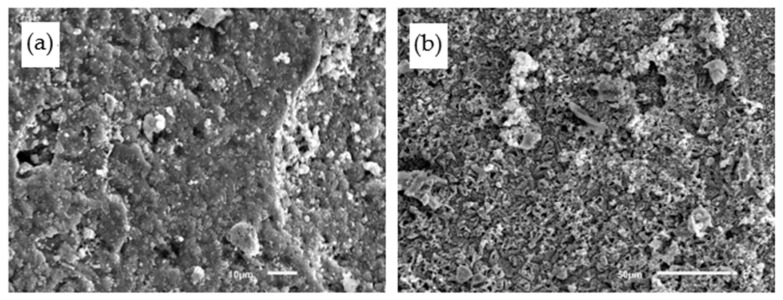
Microstructures of 8% FAG PCPC. (**a**) Without wet-dry cycle and (**b**) with 12 wet-dry cycles in acid rain solution.

Figure 13 shows the microstructures of 8% FAG PCPC. No obvious microcrack is found in 8% FAG PCPC, as shown in Figure 13a. The enhancement of the strength and the acid rain resistance of PCPC due to adding FAG can be attributed to the following mechanisms. The first one is the micro-crack shield, which can reduce the stress in the fracture process area. The second one is the crack bridging. Usually, cracks will bypass the high-strength aggregates and propagate in the cement matrix. At this time, the aggregate act as a bridge for the crack to cross. The macro-crack has a certain degree of closure due to the release of the residual stress. Therefore, fine aggregates provide an opportunity for the crack bridging [59]. Moreover, the relative movement of the aggregate particles between the crack surfaces requires energy, which improves the fracture resistance [60,61,62]. In addition, the usage of the fine aggregates also increases the bond points between coarse aggregates, which improves the strength of PCPC [63,64]. Zaetan et al. [65] found a similar result and pointed out that the increase in compressive strength was due to the filler effect of fine aggregates. The fine aggregates could improve the gradation of single size aggregates and thus alter the properties of concrete accordingly. After, the microstructures of 8% FAG PCPC with 12 wet-dry cycles in acid rain solution are still dense and no obvious microcrack occurs, as shown in Figure 13b. Therefore, FAG improves the acid rain resistance of PCPC. The addition of FAG is a good choice for making high-strength and durable PCPC [50]. Geopolymer [66] and nanoadditives [67] will also be considered to design PCPC in the future work.

## 5. Conclusions

The properties of PCPC subjected to wet-dry cycles in acid rain solution were investigated and the following conclusions are obtained.

For PCPC with 12 wet-dry cycles in acid rain solution:

(1) The compressive strength and the flexural strength of control PCPC are decreased by 30.7% and 40.8%, respectively.
(2) The final compressive and flexural strength of 4% SF PCPC are 6.9% and 25.4% greater than that of the control PCPC, respectively.
(3) The final compressive and flexural strengths of 8% FAG are higher 30.3% and 72.3% than that of the control PCPC, respectively.
(4) The wear loss of PCPC is decreased by 58.8% and 81.9% when 4% SF and 8% FAG are used to replace part of cement and coarse aggregates, respectively.
(5) The addition of 4% SF and 8% FAG improves the acid rain resistance of PCPC. 4% SF increases the water permeability, but FAG reduces the water permeability of PCPC.

## Figures and Tables

**Table 1:** Chemical compositions of cement (%).

SiO_2_	Al_2_O_3_	Fe_2_O_3_	CaO	MgO	SO_3_	Na_2_O	K_2_O	TiO_2_
23.1	7.11	3.69	57.57	2.20	2.67	2.16	0.73	0.33

**Table 2:** Mixing proportions and void ratio of PCPC.

	Water (kg/m^3^)	Cement (kg/m^3^)	Coarse Aggregate (kg/m^3^)	Fine Aggregate (kg/m^3^)	Silica Fume (kg/m^3^)	SP (kg/m^3^)	Void Ratio (%)
Control PCPC	116	415	1453	0	0	4.15	18.5
4%SF PCPC	116	398.4	1453	0	16.6	4.15	20.1
8%FAG PCPC	116	415	1336.8	116.2	0	4.15	15.3

**Table 3:** The total number of the specimens in wet-dry cycles.

	Cube 100 mm × 100 mm × 100 mm	Prism 100 mm × 100 mm × 400 mm	Cube 150 mm × 150 mm × 150 mm
Control PCPC	27	27	3
4%SF PCPC	27	15	3
8%FAG PCPC	15	15	3

## Data Availability

The data used to support the findings of this study are included within the article.
